# Supplementary material for: Sleep quality and its predictors among waiters in upscale restaurants: A descriptive study in the Accra Metropolis
Source: PLoS One. 2020 Oct 13;15(10):e0240599. doi: 10.1371/journal.pone.0240599 (PMC7553280; doi:10.1371/journal.pone.0240599)
Supplement: S1 Questionnaire — (PDF) [file pone.0240599.s001.pdf]

QUESTIONNAIRE ASSESSING SLEEP QUALITY AND ASSOCIATED FACTORS AMONG WAITERS

SECTION A: SOCIO-DEMOGRAPHIC INFORMATION	
**Question**	**Response**
1. What is your sex?	1. Female<br>2. Male
2. How old are you?	[…….…] years
3. What is your marital status?	1. Single<br>2. Co-habiting<br>3. Married<br>4. Divorced/Separated<br>5. Widowed
4. Which religion do you practice?	1. Christianity<br>2. Islam<br>3. African Traditionalist<br>4. Other:…………….
5. What is your highest level of education?	1. Primary<br>2. JSS/JHS<br>3. SSS/SHS<br>4. Tertiary
6. Which ethnic group do you belong to?	1. Akan<br>2. Ewe<br>3. Ga/Dangme<br>4. Ewe<br>5. Mole-Dagbani<br>6. Other:…………..
7. For how long have you been working as a waiter?	1. <1 year<br>2. 1-5 years<br>3. 6-10 years<br>4. 10+ years
8. For how long have you been working as a waiter?	1. <1 year<br>2. 1-5 years<br>3. 6-10 years<br>4. 10+ years
9. What is your role in this restaurant?	1. Headwaiter<br>2. Stationed waiter<br>3. Supervisor
10. Have you ever been diagnosed with any sleep problem?	1. Yes: ……………<br>2. No

SECTION B: PROSPECTS AND CHALLENGES ASSOCIATED WITH WAITING WORK IN UPSCALE RESTAURANTS	
11. Are you positive about career success in current facility?	1. No<br>2. Yes

12. Do you foresee potential of extended work involvement with current facility?	1. No<br>2. Yes
13. Do you foresee better remuneration?	1. No<br>2. Yes
14. Do you anticipate getting an advantage for higher roles/position in current facility?	1. No<br>2. Yes
15. Which of the following challenges do you experience working in this facility?	1. Lowered self-esteem<br>2. Job insecurity<br>3. Loss of interest<br>4. Emotional exhaustion<br>5. Low motivation<br>6. Other:……………………

SECTION C: SUBSTANCE USE, DEPRESSION, ANXIETY AND STRESS SCALE

0 Did not apply to me at all
1 Applied to me to some degree, or some of the time
2 Applied to me to a considerable degree or a good part of time
3 Applied to me very much or most of the time

**Statement**	**0**	**1**	**2**	**3**
16. I found it hard to wind down				
17. I was aware of dryness of my mouth				
18. I couldn't seem to experience any positive feeling at all				
19. I experienced breathing difficulty (e.g. excessively rapid breathing, breathlessness in the absence of physical exertion)				
20. I found it difficult to work up the initiative to do things				
21. I tended to over-react to situations				
22. I experienced trembling (e.g. in the hands)				
23. I felt that I was using a lot of nervous energy				
24. I was worried about situations in which I might panic and make a fool of myself				
25. I felt that I had nothing to look forward to				
26. I found myself getting agitated				
27. I found it difficult to relax				
28. I felt down-hearted and blue				
29. I was intolerant of anything that kept me from getting on with what I was doing				
30. I felt I was close to panic				
31. was unable to become enthusiastic about anything				
32. I felt I wasn't worth much as a person				
33. I felt that I was rather touchy				
34. I was aware of the action of my heart in the absence of physical exertion (e.g. sense of heart rate increase, heart missing a beat)				
35. I felt scared without any good reason				

36. I felt that life was meaningless				
**Substance use within the last 30 days**			**Yes**	**No**
37. Did you consume caffeine beverage or products?				
38. Did you consume alcoholic beverage?				
39. Did you smoke cigarette smoking?				
40. Did you smoke or consume marijuana?				
41. Did you use any non-prescription drug?				

**SECTION D: PITTSBURGH SLEEP QUALITY INDEX SCALE**<br>0 Not during the past month<br>1 Less than once a week<br>2 Once or twice a week<br>3 Three or more times a week				
42. When have you usually gone to bed?			………..	
43. How long (in minutes) has it taken you to fall asleep each night?			………..	
44. What time have you usually gotten up in the morning?			………..	
45. A. How many hours of actual sleep did you get at night?<br>B. How many hours were you in bed?			………..<br>…………	
**Statement**	**0**	**1**	**2**	**3**
46. During the past month, how often have you had trouble sleeping because you				
47. Cannot get to sleep within 30 minutes				
48. Wake up in the middle of the night or early morning				
49. Have to get up to use the bathroom				
50. Cannot breathe comfortably				
51. Cough or snore loudly				
52. Feel too cold				
53. Feel too hot				
54. Have bad dreams				
55. Have pain				
56. Other reason(s), please describe, including how often you have had trouble sleeping because of this reason(s): …………………………………………………………..				
57. During the past month, how often have you taken medicine (prescribed or "over the counter") to help you sleep?				
58. During the past month, how often have you had trouble staying awake while driving, eating meals, or engaging in social activity?				
59. During the past month, how much of a problem has it been for you to keep up enthusiasm to get things done?				
60. During the past month, how would you rate your sleep quality overall?	Very Good	Fairly Good	Fairly Bad	Very Bad

Thank you.
