# Supplementary material for: Sleep quality and its predictors among waiters in upscale restaurants: A descriptive study in the Accra Metropolis
Source: PLoS One. 2020 Oct 13;15(10):e0240599. doi: 10.1371/journal.pone.0240599 (PMC7553280; doi:10.1371/journal.pone.0240599)
Supplement: Attachment — Submitted filename: Response to Reviewers.docx [file pone.0240599.s003.docx]

**Response to Reviewers Comments raised on the Manuscript:** PONE-D-20-07362

**Sleep quality and its predictors among waiters in upscale restaurants: A descriptive study in Accra Metropolis**

Please find below our step-by-step response to the comments raised:

1. Journal Requirements

**Comment 1:** Please ensure that your manuscript meets PLOS ONE's style requirements, including those for file naming.

**Response: The manuscript has been reformatted to meet PLOS ONE’s style requirements.**

**Comment 2:** In your Methods section, please provide additional information about the participant recruitment method and the demographic details of your participants. Please ensure you have provided sufficient details to replicate the analyses such as: a) the recruitment date range (month and year), b) a description of any inclusion/exclusion criteria that were applied to participant recruitment, c) a table of relevant demographic details, d) a statement as to whether your sample can be considered representative of a larger population, e) a description of how participants were recruited, and f) descriptions of where participants were recruited and where the research took place.

**Response: The methods section has been revised appropriately (See page 7 to 8). A table of relevant demographic details is presented as part of the Results section (See page 10).**

**Comment 3:** Please include additional information regarding the survey or questionnaire used in the study and ensure that you have provided sufficient details that others could replicate the analyses. For instance, if you developed a questionnaire as part of this study and it is not under a copyright more restrictive than CC-BY, please include a copy, in both the original language and English, as Supporting Information. Moreover, please include more details on how the questionnaire was pre-tested, and whether it was validated.

**Response: Additional information on study questionnaire, pre-testing and validation has been provided (see page 8). The research instrument has also been provided as an additional file.**

**Comment 4:** Please correct your reference to "p=0.000" to "p<0.001" or as similarly appropriate, as p values cannot equal zero.

**Response: The p-values have been corrected to p<0.001 as recommended by the reviewer (See page 18).**

**Comment 5:** In your Conclusions and Abstract sections, please ensure that only conclusions that can be directly drawn from the data here presented are reported. For example, it is not clear how the data shown in the present analysis can support the statements at lines 268 to 273.

**Response: The conclusions in the revised manuscript have been linked to the key findings of the study (See pages 22) as the hospitality industry employs a substantial proportion of the general population.**

**Comment 6:** We suggest you thoroughly copyedit your manuscript for language usage, spelling, and grammar. If you do not know anyone who can help you do this, you may wish to consider employing a professional scientific editing service.

**Response: The manuscript has been copyedited by an expert, Mr. Ebenezer Agbaglo who is an English Language expert. He has been duly acknowledged in the revised manuscript (See page 23).**

**Comment 7:** We note that you have stated that you will provide repository information for your data at acceptance. Should your manuscript be accepted for publication, we will hold it until you provide the relevant accession numbers or DOIs necessary to access your data. If you wish to make changes to your Data Availability statement, please describe these changes in your cover letter and we will update your Data Availability statement to reflect the information you provide.

**Response: Contrary to the initial data availability statement. We have now provided the data used for our analysis as an additional file. Our data availability has, thus, also been changed to reflect same.**

**Comment 8:** We note that [Figure(s) 1] in your submission contain [map/satellite] images which may be copyrighted. All PLOS content is published under the Creative Commons Attribution License (CC BY 4.0), which means that the manuscript, images, and Supporting Information files will be freely available online, and any third party is permitted to access, download, copy, distribute, and use these materials in any way, even commercially, with proper attribution. For these reasons, we cannot publish previously copyrighted maps or satellite images created using proprietary data, such as Google software (Google Maps, Street View, and Earth).

**Response: Figure 1 is not copyrighted. The source publication has, however, been duly cited in our revised manuscript.**

2. Reviewer #1

**Comment:** The authors should add in the Limitations section that also the level of physical activity, which was not assessed in this study, could also be a predictor of sleep quality.

**Response: This has been done (See page 21).**

3. Reviewer #2

**Comment 1:** First page of introduction is just about growing fast food restaurants and the importance of competition between restaurant and so on (which is not your main goal). The introduction needs a revision in order to highlight the sleep quality more and the factors affecting that. The reasons why this happens might to summarize in a single paragraph. Please summarize the introduction.

**Response: The introduction has been revised as recommended by the reviewer (See pages 4-6).**

**Comment 2:** How this study would help and be of importance? Please mention in the introduction more clearly and in brief.

**Response: The importance of this study has been presented in the introduction (See page 6).**

**Comment 3:** The last two sentences of introduction are saying two different things. Mention the overall and primary goal of your study.

**Response: The two sentences have been reconciled to present the primary goal of the study (See page 6).**

**Comment 4:** There is a main point regarding sampling method. How did you choose the restaurants (what kind of sampling methods)? The result can generalize to the whole population of waiters if we use probability sampling methods. But it is not clear how you chose restaurants.

**Response: The sampling method used in selecting the six restaurants and how it was carried out has been explained in the revised manuscript (See page 7).**

**Comment 5:** Please mention the full phrases of abbreviations in the last raw of tables. What are COR and AOR in the table 4, for example.

**Response: The full phrases of abbreviation (COR and AOR) in table 4 has been stated in the last raw of the table (see page 18) as well as in the Analysis sub-section (see page 9).**

**Comment 6:** What statistical analysis is used (the results in Table 4)? Mention in the statistical analysis part (just mentioned in the abstract). As you used logistic regression, so mention odds ratio (OR) in the statistical analysis and also use the abbreviation of OR in the manuscript. Why most of the cells are empty in the Table 4?

**Response: The statistical analysis used has been stated. In the logistic regression analyses, only variables that were significant in the Chi square test were used that is why some of the cells are empty. This has been stated in the Analysis sub-section (see page 9).**
